# Interactions between rostral and caudal cortical motor areas in the rat

**DOI:** 10.1152/jn.00760.2014

**Published:** 2015-04-08

**Authors:** J. E. Deffeyes, B. Touvykine, S. Quessy, N. Dancause

**Affiliations:** ^1^Département de Neurosciences, Faculté de Médecine, Université de Montréal, Montréal, Québec, Canada; and; ^2^Groupe de recherche sur le système nerveux central, Faculté de Médecine, Université de Montréal, Montréal, Québec, Canada

**Keywords:** arm, motor cortex, motor evoked potential, premotor

## Abstract

In rats, forelimb movements can be evoked from two distinct cortical regions, the rostral (RFA) and the caudal (CFA) forelimb areas. RFA and CFA have numerous reciprocal connections, and their projections reach several common targets, which allows them to interact at multiple levels of the motor axis. Lesions affecting these areas result in profound and persistent deficits, supporting their essential role for the production of arm and hand movements. Whereas rats are widely used to study motor control and recovery following lesions, little is known as to how cortical motor areas in this model interact to generate movements. To study interactions between RFA and CFA, we used paired-pulse protocols with intracortical microstimulation techniques (ICMS). A conditioning stimulus (C) in RFA was applied simultaneously, or before a test stimulus (T) in CFA. The impact of RFA conditioning on CFA outputs was quantified by recording electromyographic signals (EMG) signals from the contralateral arm muscles. We found that stimulation of RFA substantially modulates the intensity of CFA outputs while only mildly affecting the latency. In general, the effect of RFA conditioning changed from predominantly facilitatory to inhibitory with increasing delays between the C and the T stimulus. However, inspection of individual cortical sites revealed that RFA has a wide range of influence on CFA outputs with each interstimulation delay we used. Our results show that RFA has powerful and complex modulatory effects on CFA outputs that can allow it to play a major role in the cortical control of forelimb movements.

in monkeys, several segregated forelimb representations are found in premotor areas (Dum and Strick 2002). These premotor areas have corticospinal projections and numerous reciprocal connections with the primary motor cortex (M1). Cortical interactions between premotor areas and M1 are essential to the control of the arm and hand (Davare et al. 2008; Prabhu et al. 2009). Following damage in M1, premotor areas undergo many physiological (Dancause et al. 2006b; Eisner-Janowicz et al. 2008; Frost et al. 2003) and anatomical (Dancause et al. 2005; McNeal et al. 2010) changes and compensate for the functional loss created by the lesion (Liu and Rouiller 1999). Similarly in humans, several imaging and stimulation studies have shown that the ipsilesional premotor areas can undergo functional changes and undertake new functions after stroke (Fridman et al. 2004; Seitz et al. 1998; Ward et al. 2007). Altogether, these data support that there is a reorganization of ispilesional premotor areas after injury and that plasticity in these areas is important for motor recovery.

Rats, much like primates, can perform complex arm and hand movements (Whishaw 1996), and lesions of the motor cortex greatly affect these functions (Barth et al. 1990; Gharbawie et al. 2005). The recovery of motor function following corticospinal tract injury in rats follows a pattern that is similar to what is known in primates (Whishaw et al. 1993). Accordingly, rats are widely used to study motor recovery after brain injury (Klein et al. 2012; Whishaw et al. 2008). However, in spite of the similarities between rats and primates, there also are several interspecies differences. Notably, the cortical premotor network in rats is simpler than in primates. To facilitate the interpretation and the translation of results from lesion studies in rats, we need a clear understanding of the cortical motor network and how motor areas interact to produce movements in this model.

In rats, two distinct regions in the agranular frontal cortex, the caudal (CFA) and the rostral (RFA) forelimb areas, send projections to the cervical enlargement of the spinal cord (Li et al. 1990; Rouiller et al. 1993). Intracortical microstimulation (ICMS) studies have revealed that in each of these two areas movements of the shoulder, elbow, forearm, wrist, and digits are intermingled to form a mosaic (Donoghue and Wise 1982; Hicks and D'Amato 1977). CFA extends over a much larger territory than RFA (Neafsey et al. 1986) and is located where the largest layer V neurons are found (Wang and Kurata 1998). Typically, the two forelimb areas are isolated by representations of the trunk, neck, and vibrissa (Neafsey et al. 1986; Neafsey and Sievert 1982).

CFA is generally accepted as the equivalent of M1 of primates, and there is strong evidence to suggest that the RFA would be the equivalent of a premotor area (Neafsey et al. 1986; Rouiller et al. 1993; Wise et al. 1979). Both CFA and RFA have neurons with activity tightly coupled to movement production (Hyland 1998), and the two areas have numerous reciprocal connections (Rouiller et al. 1993). Thus, independent of their hierarchical status, based on their pattern of connections, RFA and CFA are clearly two key nodes of the cortical network involved in the control of forelimb movements in rats. As such, their activity and outputs are likely to be tightly coordinated to give rise to muscle activity. However, we currently have very little information on how these two areas interact for the production of motor outputs. In monkeys, premotor areas can modulate M1's outputs to forearm and hand muscles (Cerri et al. 2003; Prabhu et al. 2009; Shimazu et al. 2004). A crucial question regarding RFA is whether it modulates CFA outputs similarly to premotor areas in primates. This information would increase our understanding of the role of RFA in motor control and help to interpret how changes in RFA after lesion can be compared with reorganization of premotor areas in humans after brain injury.

The goal of the present study was to investigate the modulatory effects of RFA on CFA outputs. To this end, we used ICMS in the context of a paired-pulse protocol (Cerri et al. 2003). This paired-pulse protocol is similar to that used in humans (with transcranical magnetic stimulations) and in nonhuman primates (with ICMS as in the present work), allowing ready comparison with previous studies. Our results show that RFA produces strong modulation of CFA output. Similarly to premotor areas, RFA can use these powerful cortical interactions to play an essential role in the cortical control of dexterous arm and hand movements and contribute to motor recovery following lesions.

## MATERIALS AND METHODS

### Subjects

Seven purpose-bred adult Sprague-Dawley rats (Charles River, St. Constant QC, Canada) with a mean weight of 330 ± 45 grams were used in this study. Rats were individually housed and supplied with food and water ad libitum before surgery. The experimental protocol followed the guidelines of Canadian Council on Animal Care and was approved by the Comité de Déontologie de l'Expérimentation sur les Animaux of the Université de Montréal.

### Surgical Procedure

Anesthesia was induced with an initial intraperitoneal injection of 80 mg/kg of ketamine hydrochloride (Ketaset; Pfizer, New York, NY) and maintained during the surgical procedures with ∼2% isoflurane (Furane; Baxter, Deerfield, IL) in 100% oxygen. Animals received dexamethasone (1 mg/kg im) to reduce inflammation and saline injections (5 ml/kg/h sc) to maintain hydration over the experimental procedure. Animal body temperature was maintained near 36.5°C throughout the surgery with a homeothermic blanket (Harvard Apparatus, Holliston, MA). Blood oxygen saturation was monitored by means of a pulse oximetry sensor (Nellcor Puritan Bennett, Mansfield, MA).

Insulated, multistranded wires (Cooner Wire, Chatsworth, CA) were implanted intramuscularly to record electromyographic (EMG) signals. In one rat, only the extensor digitorum communis (EDC) and palmaris longus (PL) in the arm contralateral to the stimulated hemisphere were implanted. In the six other animals, the EDC, PL, biceps brachii (BB), and triceps brachii (TB) were implanted. Accurate placement of the EMG wires was confirmed by electrical stimulation of the muscle using the implanted wires and observing the evoked movements. A craniotomy and durectomy were performed to expose the two forelimb representations of the motor cortex. The opening was covered with mineral oil to protect the cortex. An incision of the cisterna magna was made to drain cerebral spinal fluid to lower intracranial pressure and reopened as needed when cerebral swelling was observed.

### ICMS Mapping Techniques

To locate CFA and RFA, we conducted cortical mapping using ICMS trains as in our previous studies (Mansoori et al. 2014). A high-resolution digital photograph was taken, and the locations of electrode penetrations were recorded on the photograph relative to the visible cortical vasculature with an image-processing software (Canvas, version 11; ACD Systems, Seattle, WA). Gas anesthesia was switched to ketamine (∼5–10 mg·kg^−1^·10 min^−1^ ip) at this time. A glass insulated, tungsten electrode (∼1 MΩ; FHC, Bowdoin, ME) was used for ICMS. At each penetration site, the electrode was lowered to a depth of ∼1,550 μm, corresponding to layer V, using a microdrive (model 2662; David Kopf Instruments, Tujunga, CA). A programmable pulse generator (Master-8; A.M.P.I., Jerusalem, Israel) triggered a constant-current stimulus isolator (model B51-2; BAK, Mount Airy, MD). ICMS mapping was done with trains of 13 cathodal square pulses, each 0.2 ms wide, at 300 Hz. Trains were delivered at 1 Hz. At each penetration site, we recorded the response evoked at threshold current intensity (visible movement with 50% of stimulus trains), with currents <80 μA. If no movement was visible at 80 μA, the site was considered as nonresponsive. ICMS mapping was continued using a minimum electrode interpenetration distance of 333 μm until several cortical sites in CFA and RFA were identified.

### Paired-Pulse Stimulation and EMG Recording

**Fig. 1.**
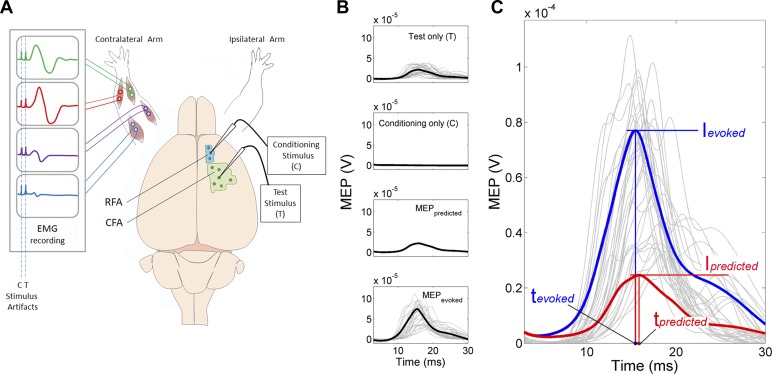
Experimental setup and data analyses. *A*: schematic showing the experimental setup. Multistranded wires were implanted in the extensor digitorum communis (green), palmaris longus (red), biceps brachii (purple), and triceps brachii (blue) bilaterally to record electromyographic (EMG) signals. Intracortical microstimulation (ICMS) trains were used to locate the caudal forelimb area (CFA, green) and rostral forelimb area (RFA, blue). Each dot in the map shows a hypothetical ICMS stimulation location. The conditioning (C) electrode was placed in RFA and the test (T) electrode in CFA. Once electrodes were in place, we recorded a stimulation protocol that included 7 stimulation conditions [C electrode only, T electrode only, or both electrodes, using one of 5 different interstimulation intervals (ISIs)]. We collected 300 trials/condition delivered at 3 Hz. Data for each condition were recorded in three blocks of 100 trials, and the stimulation condition of subsequent blocks was randomized. *B*: examples of motor evoked potential (MEP) resulting from cortical stimulations. *Inset* on *top* shows the evoked MEP from 35 individual trials (light gray) and the average response (black) when only the T stimulation in CFA was applied. *Inset* on *bottom* shows the same when only the C stimulation in RFA was applied. Because the intensity of the C stimulation was purposely set below threshold, individual trials and the average response are all close to zero. However, to account for any potential small MEP that could have been present after multiple C stimulations, we calculated an average predicted paired-pulse MEP (aMEP_predicted_). The aMEP_predicted_ (3rd *inset*) is the linear summation of the MEP evoked by the T only and the C only. Finally, the *inset* on *bottom* shows the average evoked paired-pulse MEP (aMEP_evoked_) recorded when C and T were stimulated in the same trial. An aMEP_evoked_ was calculated for each ISI used in a protocol. *C*: a first analysis was conducted on the average response. The aMEP_evoked_ (blue) was compared with the aMEP_predicted_ (red). The peak intensities (I_evoked_ and I_predicted_) and latencies (t_evoked_ and t_predicted_) were used to calculate ΔI and Δ*t*. A second analysis was based on the individual trials (see materials and methods).

The two electrodes used for the paired-pulse stimulation were single wire insulated tungsten electrodes with an impedance of ∼80 kΩ. The conditioning (C) electrode was positioned in RFA and the test (T) electrode in CFA with two independent micromanipulators (Fig. 1). Both electrodes were positioned at a depth of ∼1,550 μm, and both C and T stimulations were cathodal single square pulses of 0.2 ms duration. The intensity for the C and T stimulations was determined online, based on evoked EMG activity. The current intensity for the C stimulation was set at 75% of the EMG threshold. If evoked EMG activity was present in more than one muscle, the muscle with the lowest threshold was used to determine the desired intensity. If no EMG response could be observed with up to 300 μA, this intensity was used for the C stimulation. Values of T stimulation intensities in the CFA were typically set ∼1.25 × threshold. However, for a few sites, the evoked EMG response was too small or too big at this intensity. Thus, we adjusted the intensity to reliably evoke clear, submaximal EMG responses.

Once the electrodes were in position and intensities for the C and T stimulations established, a paired-pulse stimulation protocol was initiated. In different trials, current could be delivered with the C electrode only, the T electrode only, or with both electrodes using one of five different interstimulation intervals (ISIs) (total of 7 different conditions). In paired-pulse conditions, C and T were either delivered simultaneously (ISI0) or with C preceding T by 2.5 (ISI2.5), 5 (ISI5), 10 (ISI10), or 15 (ISI15) ms. For each of the seven stimulation conditions, we collected 300 trials delivered at 3 Hz. Data for each condition were recorded in three blocks of 100 trials, and the order of the blocks was randomized.

Paired-pulse stimulation and EMG data recording was controlled with data acquisition software custom designed for this procedure (Tucker Davis Technologies, Alachua, FL). EMG signals were recorded at 12.2 kHz for each channel. Raw EMG data were stored for offline processing.

### Electromyographic Data Analysis

Data were analyzed offline using custom written MatLab (version R2013a; Natick, MA) code. First, the continuous EMG data were separated into individual trials and aligned to stimulation time stamps. The baseline data were collected in a 35-ms window before the first stimulus. The motor evoked potential (MEP) was collected in a window of 3–30 ms after the end of the stimulation. The EMG signal was then full-wave rectified and band pass filtered (2–100 Hz). Next, we analyzed the effect of RFA conditioning on the MEP peak intensity and peak latency. For both parameters, we conducted two analyses. A first analysis was done based on the average response from all trials for each condition, and a second analysis was performed on individual trials for each condition.

#### Effect of RFA conditioning on MEP based on average responses.

To perform this first analysis, for every protocol, the MEPs of all trials were averaged for each condition. We examined the data recorded from each muscle individually and verified that the T stimulation alone evoked a clear MEP. If the average MEP peak intensity resulting from the T stimulation alone was not greater than three SDs above the average baseline activity for this condition, we excluded the data for that muscle from subsequent analyses. In 13 out of 26 protocols, the T stimulation alone did not result in a clear MEP in one of the recorded muscles (TB), and data from this muscle for these protocols were discarded.

An average predicted paired-pulse MEP (aMEP_predicted_) was calculated as the linear summation of the MEPs generated by the T stimulus alone and the C stimulus alone (Fig. 1*B*). Because the target current intensity for the C stimulus was a subthreshold value, the major contribution to aMEP_predicted_ was from the T stimulation. However, the aMEP_predicted_ was calculated to account for any potential small EMG response from the C stimulation that may have occurred over many trials. Next, an average evoked paired-pulse MEP (aMEP_evoked_), when both C-T stimulations were applied, was calculated for each of the five ISIs.

##### AVERAGE MODULATION OF PEAK INTENSITY.

For the aMEP_predicted_ and the aMEP_evoked_, peak intensity relative to the prestimulus baseline activity was determined by subtracting the baseline value from the aMEP peak intensity value (Fig. 1*C*). The modulation of aMEP peak intensity (ΔI), or the average effect of RFA conditioning on the MEP peak intensity, was calculated as the difference between the peak intensities of the aMEP_evoked_ and the aMEP_predicted_, normalized by peak intensity of the aMEP_predicted_, according to the following formula:

$$\text{Modulation}\;\text{of}\;\text{peak}\;\text{intensity}\left(\Delta \text{I}\right)=\frac{I_{\text{evoked}}-I_{\text{predicted}}}{I_{\text{predicted}}}$$

where I_evoked_ is the peak intensity of the aMEP_evoked_ for a given ISI, and I_predicted_ is the peak intensity of the aMEP_predicted_. We normalized ΔI to aMEP_predicted_ to provide a measure that would facilitate the comparison of the effects across different muscles and animals.

For each paired-pulse protocol, ΔI was calculated for all five ISIs. If ΔI is positive, it indicates that the peak intensity of the aMEP_evoked_ was greater than the peak intensity of the aMEP_predicted_, indicating a facilitation of the CFA motor outputs by the conditioning of RFA. If ΔI is negative, the peak intensity of the aMEP_evoked_ was smaller than the peak intensity of the aMEP_predicted_, indicating a suppression of the CFA motor output by the conditioning of RFA.

##### AVERAGE MODULATION OF PEAK LATENCY.

To study the modulatory effect of RFA conditioning on the latency of MEPs, we used peak latency values (Fig. 1*C*). We preferred peak latency to onset because algorithms seeking MEP onset had more variability because of noise. Whereas analyses of peak intensities are possible when the average response showed no or little evoked MEP (i.e., accurate value ∼0 V peak intensity), the identification of accurate peak latencies is unreliable in these cases. Thus, for the analyses of latency, we excluded the conditions where the average peak intensity of the aMEP_evoked_ at an ISI was not greater than three SDs above the average baseline activity before stimulation for this condition. Based on this criterion, 54 aMEP_evoked_ of the 395 were removed from the latency analysis. For the remaining data, we calculated the difference between the latency of the peaks of the aMEP_predicted_ and the aMEP_evoked_ (Δ*t* = t_evoked_ − t_predicted_) (Fig. 1*C*). Accordingly, Δ*t* is negative if the peak of MEP_evoked_ was earlier than the one of MEP_predicted_ and positive if the peak of MEP_evoked_ was later than the one of MEP_predicted_.

We used the ΔI and Δ*t* values obtained at each ISI to determine the general effect of RFA conditioning on CFA outputs across all cortical sites from which data were collected. To do so, we performed repeated-measures ANOVA on ΔI and on Δ*t* values using the factor ISI (SPSS software, version 21.0; IBM, Armonk, NY). The Greenhouse-Geisser correction to degrees of freedom was used when Mauchley's test indicated that sphericity could not be assumed (*P* < 0.05). Only the three muscles with sufficient data were included in the ANOVA (EDC, PL, BB; MEPs were more rarely observed in TB, see results). A first ANOVA was conducted with the data of all three muscles, and then three separate ANOVAs were conducted, one for each muscle. Because a total of four ANOVAs was performed, we set *p*_critical_ at 0.0125 following a Bonferroni correction. If a significant main effect of ISI was found in the ANOVA, the relationship between ΔI or Δ*t* and the ISI was examined with a linear contrast analysis.

#### Effect of RFA conditioning on MEP based on single trial responses.

One issue with ΔI and Δ*t* values is that for each protocol (i.e., a pair of RFA and CFA cortical stimulation sites) only one value is obtained for the aMEP_predicted_ and one value for the aMEP_evoked_ with each ISI. Thus, for a given protocol, no statistical analysis can be done to evaluate if the RFA conditioning significantly affected the peak intensity or latency at different ISIs. To address this issue, we conducted a second analysis for which the peak MEP was identified on individual trials. To account for the potential effect of the C stimulations in this analysis, single trials with T stimulation only and single trials with C stimulation only were randomly matched and combined to create predicted trials. A population of peak intensity and latency values was then obtained from the predicted single trials (sMEP_predicted_) and for each ISI (sMEP_evoked_). We then compared if the population of peak intensity and latency values of the sMEP_evoked_ at each of the five ISIs was different from the ones of the sMEP_predicted_ with a Wilcoxon rank sum analysis.

We recorded 26 protocols, in which we tested five different ISIs and recorded two or four muscles (total of 460 possible comparisons). For the analysis of the peak intensity, we had 13 protocols in which the T stimulation alone did not result in a clear MEP in one muscle, making the comparison of the MEPs with the five ISIs impossible (65 comparisons discarded; see above). Thus, the Wilcoxon rank sum analysis on the peak intensity was performed on a total of 395 comparisons. Using a familywise error rate Bonferroni correction for multiple comparisons, we set *p*_critical_ = 0.05/395 = 1.27 × 10^−4^.

As mentioned above, for latency analyses, we also had some instances where very little or no MEP was evoked by the stimulation of C-T at different ISIs, rendering the identification of a reliable MEP peak latency impossible on these conditions as well (54 cases). Thus, the Wilcoxon rank sum analysis on the latency was performed on a total of 341 comparisons. Using a familywise error rate Bonferroni correction for multiple comparisons, we set *p*_critical_ = 05/341 = 1.47 × 10^−4^.

#### Relation between the modulation of MEP peak intensity and latency.

Finally, for all protocols and ISIs for which we were able to get a Δ*t* value (*n* = 341; see above), we analyzed relationships between ΔI and Δ*t*. We conducted regressions for each muscle separately. Because data for each ISI are arguably not independent (evoked by the stimulation of the same electrode locations), we used Linear Mixed Effects models, correcting for repeated measurements across ISIs to examine the slope of the regression for ΔI and Δ*t* (SPSS software, version 21.0; IBM). Because a total of four regressions were conducted, a familywise Bonferroni correction for multiple comparisons was applied to the significance level, and *p*_critical_ was established at 0.0125 (0.05/4).

## RESULTS

**Fig. 2.**
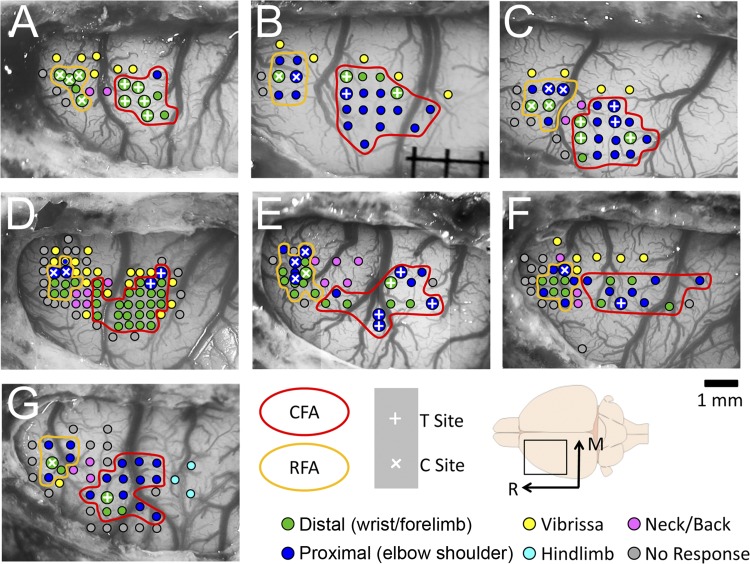
Location of cortical stimulation sites used for the paired-pulse protocols. Shown are the ICMS data collected in the 7 animals included in the study. In 6 animals (*A*–*F*), the stimulations were done in the left hemisphere, and, in 1 animal (*G*), they were done in the right. Each colored dot overlaid on the cortex is a site at which ICMS trains were delivered. Movement categories evoked at threshold current intensity are color coded. Typically, movements were evoked at lower current intensity in the CFA, and neck and vibrissa representations separated the CFA and RFA. Once the two forelimb areas were located, the electrodes were placed at cortical locations from which movements could be evoked with relatively low current intensities with ICMS trains. White × signs show the locations from which the C stimuli were delivered, and white + signs show locations used for the T stimulations. In 4 animals (*A*–*C*, *E*) some sites were used with more than one partner. M, medial; R, rostral. Scale bar = 1 mm.

A total of 26 paired-pulse protocols were conducted in seven animals. Each protocol tested the interactions between an RFA and a CFA cortical site. Figure 2 shows the ICMS mapping results in all seven animals and the cortical position of the C and T electrodes used to conduct the paired-pulse protocols. In each animal, a total of one to eight protocols were recorded (Table 1). The shortest distance between the C and T electrodes was 1.33 mm, and the average distance was 2.38 mm. For each protocol, the two stimulation sites chosen evoked the same type of movements with ICMS trains. For some protocols, we positioned the electrodes in RFA and CFA at cortical sites where movements of wrist could be evoked with ICMS trains (15 protocols). Yet, for other protocols, we positioned both electrodes where movements of the elbow were evoked (11 protocols). Thus, in relation to ICMS mapping data, we did not evaluate the effects of sites evoking distal movements in RFA on sites evoking proximal movements in CFA or of proximal sites in RFA on distal sites in CFA. Also, the limited number of sites we collected in each animal did not allow us to systematically analyze the topographic organization of interactions between RFA and CFA.

**Table 1.** Type of modulatory effects of RFA found in each animal

	No. of Protocols	Group Facilitation	Group Inhibition	Group Opposite	Not Sig
Rat_2A*^Ŧ^	6	9	0	3	0
Rat_2B	3	7	1	4	0
Rat_2C	8	10	9	0	6
Rat_2D	2	0	4	0	2
Rat_2E	5	2	9	6	0
Rat_2F	1	3	0	0	0
Rat_2G	1	1	0	0	3

Group facilitation: cases where a rostral forelimb area (RFA) site had a facilitatory effect on the motor evoked potential (MEP) of a muscle with at least one interstimulation interval (ISI), but had no significant inhibitory effect on MEPs with any ISI; group inhibition: cases where RFA had an inhibitory effect with at least one ISI but had no significant facilitatory effect with any ISI; group opposite: cases where RFA had both significant facilitatory effects and inhibitory effects on the MEP of a muscle with different ISIs; group not sig: cases where RFA stimulation did not significantly modulate the MEPs of a muscle with any of the ISIs. *Rat nos. refer to the cortical maps shown in Fig. 2. Accordingly, the cortical map of Rat_2A is shown in Fig. 2*A*. ^Ŧ^Only extensor digitorum communis and palmaris longus muscles were implanted in this rat.

**Fig. 3.**
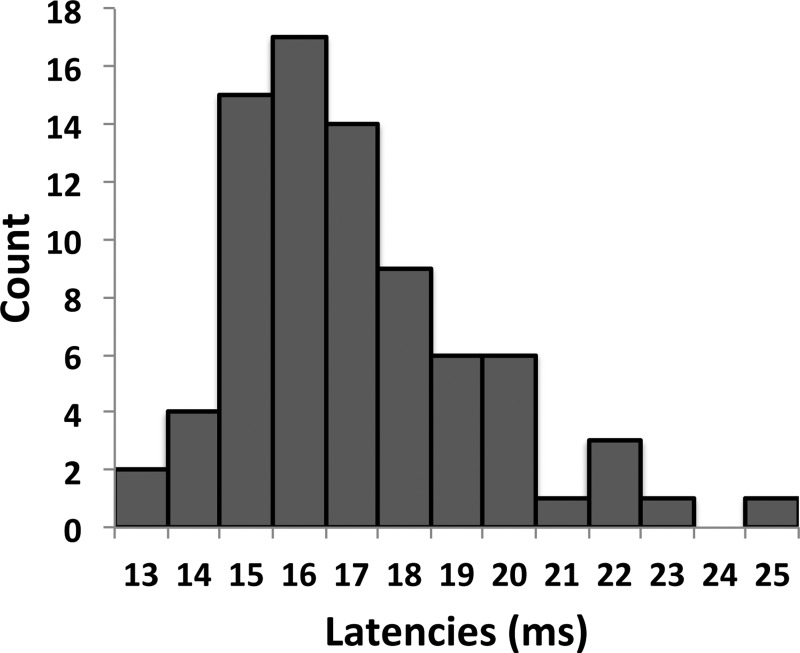
MEP peak latencies resulting from T stimulation in CFA. We evoked clear MEPs with the T stimulation from a total of 26 cortical sites. The bar graph shows how many MEP peaks we found at the different latencies. The fastest peak was found 13 ms after the T stimulation, and the slowest was found after 25 ms. The mean MEP peak latency was 17.04 ± 2.29 ms, and the median peak latency was 16.52 ms. These results support that T response is a cortically mediated response.

For each protocol, the T stimulation gave a clear MEP in at least one of the recorded muscles. Figure 3 shows the MEP peak latencies in all four muscles resulting from the 26 T stimulations sites in CFA. The peak latency values (mean ± SD) for the MEPs resulting from the T stimulation were 17.42 ± 2.10 ms for EDC, 16.83 ± 2.23 ms for PL, 16.52 ± 2.32 ms for BB, and 17.90 ± 2.46 ms for TB. The average peak latency across muscles was 17.04 ± 2.29 ms, and the median peak latency was 16.52 ms. A one-way ANOVA found no significant differences of latencies between muscles (*F* = 0.43, *P* = 0.74).

**Fig. 4.**
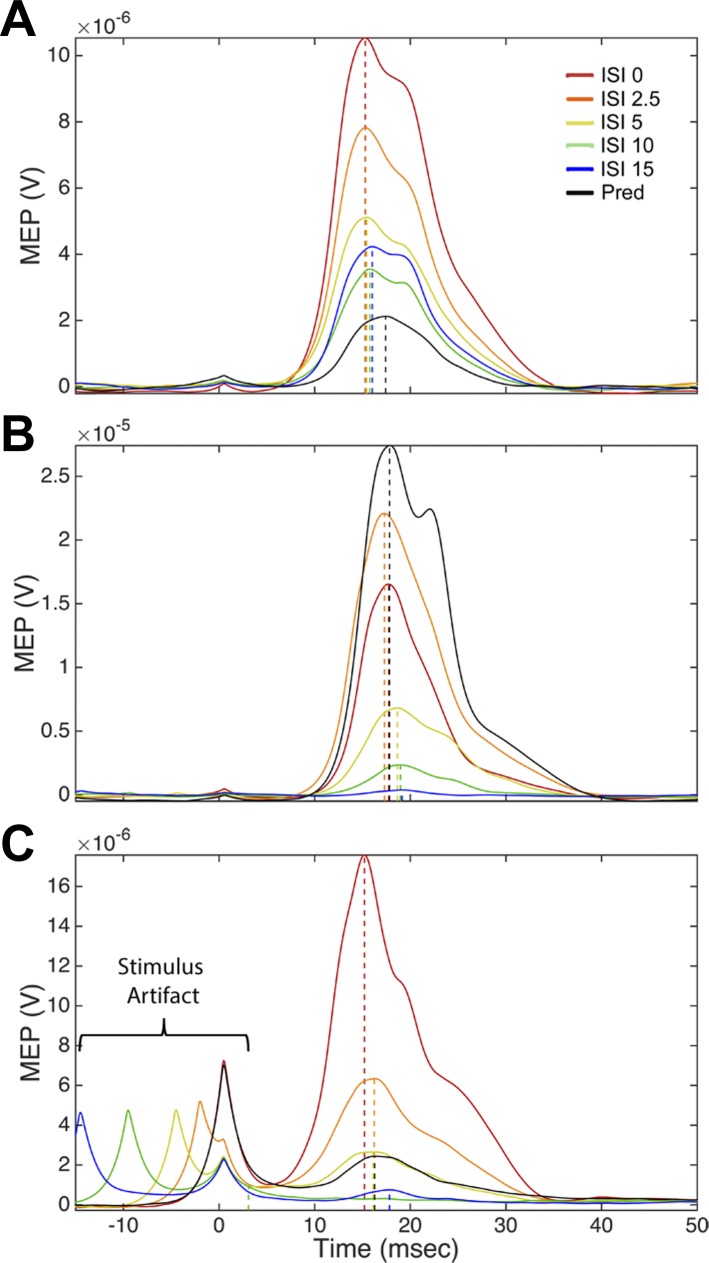
Examples of modulation of MEP resulting from RFA conditioning. Data shown are MEPs of the extensor digitorum communis (EDC) from different stimulation protocols (cortical locations). The aMEP_predicted_ (sum of T and C) is shown in black and aMEP_evoked_ with the different ISIs in color (see legend). Broken lines show examples of aMEP_evoked_ peaks and their latencies. *A*: for some cortical sites, when the conditioning stimulation of RFA had an effect on the MEP, it was always facilitatory, regardless of the ISI. In this case, the calculation of ΔI would give positive values with all ISIs. The broken line shows that the increase of peak intensity with C and T delivered simultaneously (ISI0) was associated with a decrease of latency. *B*: for other cortical sites, when conditioning RFA had an effect, it was an inhibition of the evoked MEP, regardless of the ISI. Here, ΔI would be negative with all ISIs. The broken line shows that the decrease of peak intensity with ISI10 was associated with an increase of latency. *C*: finally, the conditioning of RFA could facilitate the evoked MEP with some ISIs and inhibit the evoked MEP with other ISIs. In this example, ISI0, paired-pulse conditions with C preceding T by 2.5 ms (ISI2.5), and paired-pulse conditions with C preceding T by 5 ms (ISI5) are all facilitatory, whereas ISI10 and ISI15 are inhibitory. Accordingly, ΔI would be positive at some ISIs and negative at others, and thus have opposite effects. Broken lines show that, with ISI0, the increase of peak intensity was associated with a decrease of latency, and with ISI15, the decrease of peak intensity was associated with an increase of latency. With ISI2.5, there was an increase of peak intensity but no effect on latency.

**Fig. 5.**
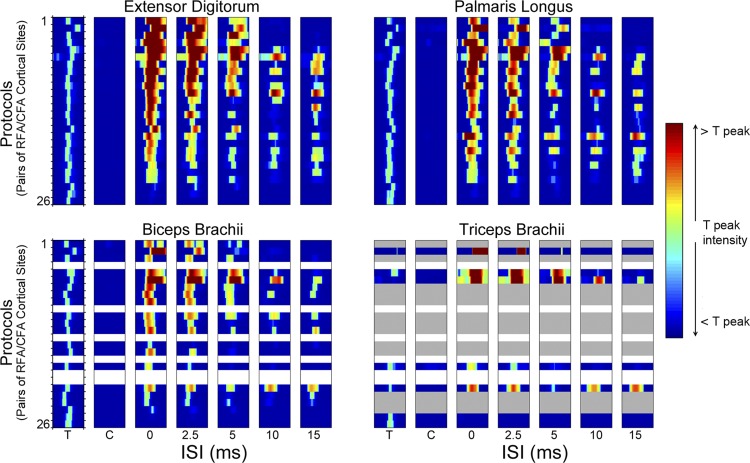
MEPs of 4 arm muscles recorded in 26 protocols. Each panel shows the data from a recorded muscle. MEPs were simultaneously recorded in the EDC (*top left*), palmaris longus (PL; *top right*), biceps brachii (BB; *bottom left*), and triceps brachii (TB; *bottom right*). Each row in each panel shows the averaged EMG data for each condition that were calculated from 3 blocks of 100 trials presented in randomized order. The protocols are ordered (from 1 to 26) based on intensity of modulation of the MEP in the EDC at ISI0 and the order kept across the different muscles. The EMG values within a row are normalized to the MEP peak intensity from the T stimulus alone. The color scale on the *right* shows the range of possible MEP values. If dark red colors are visible with an ISI, it means that the MEP evoked with C-T was much greater than the MEP evoked with T stimulation alone (e.g., *EDC protocol 1* with ISI0 and ISI2.5). If the response with an ISI shows dark blue colors, RFA conditioning had a strong inhibitory effect [e.g., *EDC protocol 1* with paired-pulse conditions with C preceding T by 10 (ISI10) or 15 ms (ISI15)]. If the response with an ISI is green, the MEP evoked with C-T was comparable to the MEP evoked with the T stimulation only (e.g., *EDC protocol 1* with ISI5). In each panel, from *left* to *right*, the MEP responses during the analyzed data window (3–30 ms after the end of stimulation) are shown for the T stimulus alone, the C stimulus alone, and C-T at the different ISIs. White rows in BB and TB correspond to protocols from the animal in which only EDC and PL were recorded. In addition, in the TB, there were 13 protocols where the T stimulation alone did not evoke EMG, and these data were discarded (gray rows). The general pattern of modulation appears consistent across muscles. This is more obvious for the EDC, PL, and BB, for which more data are available. For all 4 muscles, strong facilitations are more common with shorter ISIs (ISI0 and ISI2.5) and inhibition more common with longer ISIs (ISI10 and ISI15).

The paired stimuli gave varying responses for different protocols and with different ISIs. Figure 4 shows examples of modulation of aMEP_evoked_ with different ISIs for the EDC muscle. In some protocols, when the conditioning of RFA affected the MEP, it resulted in an increase of the MEP compared with the response from T stimulation alone, regardless of the ISIs (Fig. 4*A*). In other protocols, when RFA conditioning affected the MEP, it resulted in a decrease of the MEP compared with the response from T stimulation alone, regardless of the ISIs (Fig. 4*B*). Finally, in some protocols, RFA conditioning increased the evoked MEP at some ISIs and decreased it at others (Fig. 4*C*). The entire dataset of MEPs collected is shown as a color intensity plot in Fig. 5. Typically, T stimulation produced a clear MEP response, and the C stimulus produced no response. Clear EMG responses in the TB were less frequent than in the other muscles. Because of the low number of protocols with MEPs for this muscle, we did not include it in the ANOVA.

### Effect of RFA Conditioning on MEP Peak Intensity

**Fig. 6.**
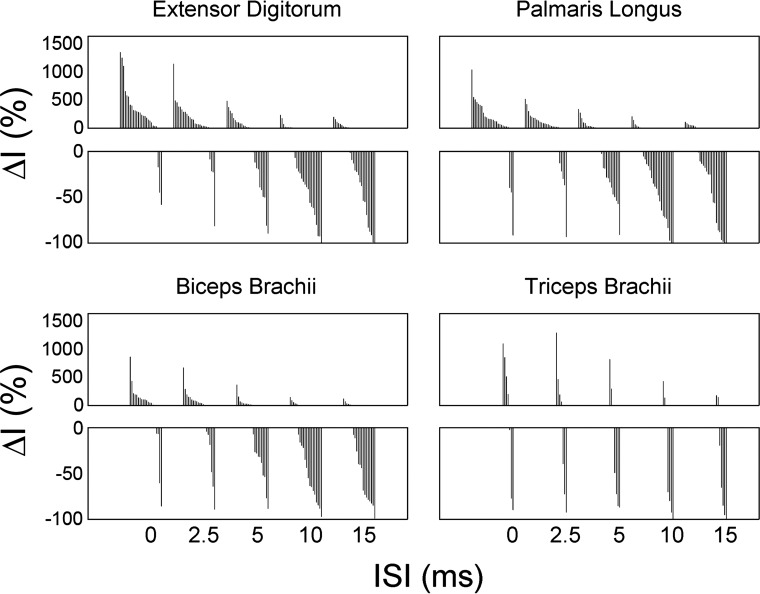
Average effect of RFA conditioning on MEP peak intensity (ΔI). ΔI values at the different ISIs for the 26 protocols (pairs of RFA and CFA stimulation sites). Data for the EDC (*top left*), PL (*top right*), BB (*bottom left*), and TB (*bottom right*) are separated. A column in the bar graphs shows the ΔI value of a protocol at an ISI. Data are sorted from highest to lowest ΔI within each ISI. Because the percent change for inhibition is limited to the range 0–100 and the facilitatory effect can be infinite, we separated the facilitatory and inhibitory effects in two plots with different scales. The facilitatory effects are shown in plot on *top* and inhibitory effects in the plot on the *bottom*. The effect of RFA conditioning on peak intensity appeared to be consistent in the different muscles. At each ISI, we found RFA sites that had both facilitatory and inhibitory effects on CFA outputs. However, at short ISIs, facilitatory effects were much more common, and, at long ISIs, inhibitory effects were predominant.

The first analysis we conducted on MEP peak intensity was based on the averaged MEPs with each ISI. Figure 6 shows the incidence of facilitatory and inhibitory ΔI values with each ISI for the entire population of protocols we recorded. When analyzing the effects across all pairs of cortical sites, it becomes clearer that most C stimulations in RFA resulted in a strong facilitatory effect on MEPs when they were delivered simultaneously (ISI0) or shortly before the T stimulation in CFA (ISI2.5). With increasing time between the stimuli, the facilitatory effects were not as strong and suppression was more frequently observed. At ISI10 and ISI15, the effect of RFA stimulation was most often inhibitory on CFA outputs. We conducted ANOVA for the three muscles from which we were able to evoke sufficient responses (EDC, PL, and BB). First, an ANOVA was conducted on the data of all three muscles together. We found that the main effect of ISI was significant (*F* = 43.39, *P* < 0.001), and the inverse linear relation between ΔI and ISI was also significant (linear contrast; *F* = 53.18, *P* < 0.001). With increasing delays between the C and T stimuli, RFA’s general modulatory effect on peak intensity progressively went from facilitatory to inhibitory. When looking at muscles individually, the effect of ISI on MEP peak intensity was also significant (EDC: *F* = 18.02, *P* < 0.001; PL: *F* = 19.80, *P* < 0.001; BB: *F* = 10.27, *P* = 0.001) as well as the linear contrast for all three muscles (EDC: *F* = 20.97, *P* < 0.001; PL: *F* = 26.75, *P* < 0.001; BB: *F* = 13.77, *P* = 0.001). Thus, the inverse relationship between the effect of RFA conditioning of MEP peak intensity and ISI was significant, regardless of the recorded muscle.

However, the modulatory effect of RFA was variable for different cortical sites (i.e., protocols). For example, whereas the average effect with ISI0 was a strong facilitation in EDC, a few RFA sites did exert an inhibitory effect on CFA outputs to this muscle (see Fig. 6). Averaging over all protocols as was done in the ANOVA above may have disregarded some interesting differences between RFA cortical sites. Therefore, we conducted a second analysis based on individual trials. sMEP_predicted_ were created by randomly combining trials with T stimulation only and C stimulation only. The peak intensity was identified in each trial to get a population of predicted peak intensity values. Similarly for each ISI, peak intensity values were identified in single trials (sMEP_evoked_). For each ISI, Wilcoxon rank sum analysis was used to test if sMEP_evoked_ peak intensity was different from sMEP_predicted_ peak intensity. Accordingly, the Wilcoxon test is performed on the individual trials for each ISI within a protocol, and this test gives one value, either facilitation, suppression, or no change.

First, we found cases where an RFA site had a facilitatory effect on the MEP of a muscle with at least one ISI, but had no significant inhibitory effect on MEPs with any ISI (Group Facilitatory; *n* = 32 or 40.5%). Second, we found cases where RFA had an inhibitory effect with at least one ISI but had no significant facilitatory effect with any ISI (Group Inhibitory; *n* = 23 or 29.1%). Third, we found cases where RFA had both significant facilitatory effects and inhibitory effects on the MEP of a muscle with different ISIs (Group Opposite; *n* = 13 or 16.5%). Finally, we found some cases where RFA stimulation did not significantly modulate the MEPs of a muscle with any of the ISIs (Not Sig; *n* = 11 or 13.9%). The number of protocols and type of responses found in each rat are shown in Table 1.

**Fig. 7.**
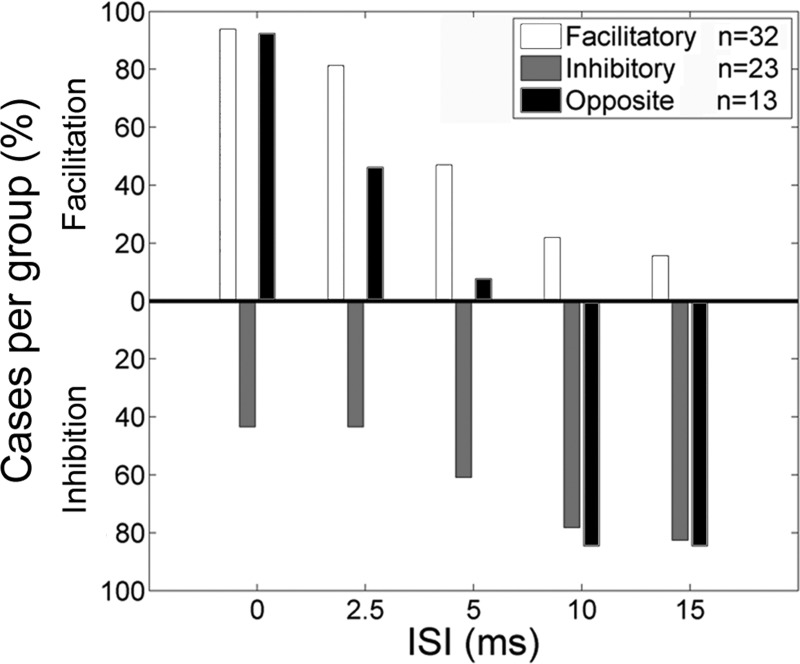
Categories of significant RFA conditioning effects on MEP peak intensity. Results of the Wilcoxon rank sum analysis for peak intensity. For a given protocol, we tested if the population of sMEP_evoked_ was significantly greater (facilitation) or smaller (inhibition) than the sMEP_predicted_ with the various ISIs. For this analysis, we combined data from all muscles. We separated the pattern of RFA modulation on CFA outputs across ISIs into 4 categories. The bar graph shows the percentage of RFA sites within each category that had a significant effect with the different ISIs. First, the most common pattern of modulatory effects on CFA output intensity resulting from RFA conditioning was a significant facilitation with at least one ISI, but no significant inhibitory effect on MEPs with any ISI (Group Facilitatory; *n* = 32). Within Group Facilitatory, most RFA sites significantly modulated CFA outputs with short ISIs, and this percentage decreased with longer ISIs. Second, we found fewer, but still many, RFA sites that significantly inhibited CFA outputs with at least one ISI but had no significant facilitatory effect with any ISI (Group Inhibitory; *n* = 23). Fewer sites from Group Inhibitory had a significant effect with short ISIs than with long ISIs. Third, some RFA sites had both significant facilitatory effects and inhibitory effects on the MEP of a muscle with different ISIs (Group Opposite; *n* = 13). With short ISIs, a large proportion of cortical sites from Group Opposite was significantly facilitatory, and no sites were inhibitory. In contrast, with long ISIs, most sites from Group Opposite were inhibitory, and none were facilitatory. Finally, we found RFA sites that did not significantly modulate the MEPs of a muscle with any of the ISIs (Not Sig; *n* = 11).

Figure 7 shows the distribution of the significant RFA effects across ISIs for each of these groups. For every ISI, the percentage of sites with a significant effect within each group was calculated. For example, out of the 32 cases in Group Facilitatory, 30 had a significant facilitatory effect with ISI of 0 ms (93.8%). For Group Facilitatory, the number of cases with a significant effect decreased with longer ISIs, and with ISI15 only 15.6% of cases had a significant effect. We found the opposite relation between RFA modulatory effect and ISI for Group Inhibitory. For this group, 43.5% of cases had a significantly inhibitory effect with ISI0 and ISI2.5. This proportion increased with longer ISIs, and, with ISI15, 82.6% of cases in Group Inhibitory had a significant effect. Finally, for Group Opposite, only facilitatory effects were significant with ISI0 (92.3%), ISI2.5 (46.2%), and ISI5 (7.7%). With ISI10 and ISI15, only inhibitory effects were significant (84.6% for both ISIs). This analysis confirmed that most cases in which RFA conditioning was facilitatory had their effects with short ISIs, and most cases in which RFA conditioning was inhibitory had their effects with long ISIs. Interestingly, all cases in which RFA had opposite effects were also facilitatory with short ISIs and inhibitory with long ISIs.

Finally, we inspected the effects of RFA conditioning on the MEPs across muscles. Only data from the six animals with all four muscles recorded were included in this analysis (20 protocols × 5 ISIs = 100 total cases). Theoretically, stimulation of an RFA site could have facilitatory effects on the MEPs of up to four muscles, have inhibitory effects on up to four muscles, or simultaneously facilitate and inhibit different combinations of muscles. In 36% of cases, RFA stimulation did not affect the MEP of any of the muscles. We found no cases where RFA conditioning simultaneously increased the MEP of one muscle and decreased the MEP of another muscle. We only found cases where RFA conditioning either had facilitatory or inhibitory effects in the recorded muscles. We found that 9% of RFA sites simultaneously facilitated three of the four muscles, 7% facilitated one or two muscles, and 6% facilitated all four muscles. For inhibitory effects, we found that 11% of RFA sites inhibited only one muscle, and 10, 9, and 5% simultaneously inhibited all four, three, and two muscles, respectively. Thus, it seems that, for both facilitation and inhibition, the effect of RFA could be targeted to a single muscle but could also be more global and affect several muscles simultaneously.

### Effect of RFA Conditioning on MEP Peak Latency

**Fig. 8.**
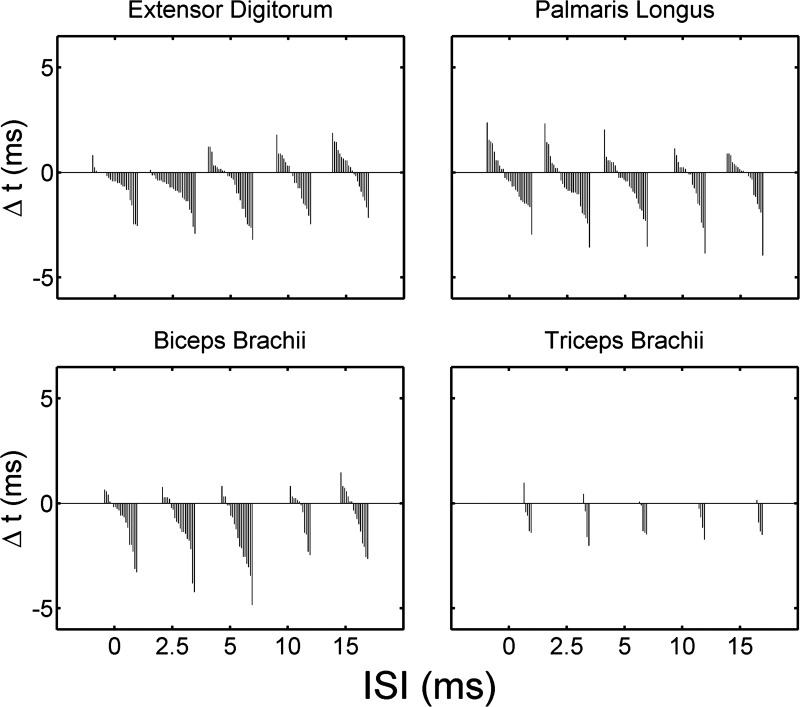
Average effect of RFA conditioning on MEP peak latency (Δ*t*). Bar graphs showing the effect of RFA conditioning on the MEP peak latency values (Δ*t*) for EDC (*top left*), PL (*top right*), BB (*bottom left*), and TB (*bottom right*). A column in the bar graphs shows the Δ*t* value of a protocol at an ISI. Data are sorted from highest to lowest Δ*t* within each ISI. For each muscle, the RFA conditioning could increase (positive values) or decrease (negative values) the latency of the aMEP_evoked_ peak compared with the aMEP_predicted_ peak. In most cases, the modulation of latency resulting from RFA conditioning was quite small. In general, RFA conditioning tended to decrease the latency of the MEPs more often with short ISIs and increase it with longer ISIs. Among the 3 muscles for which sufficient data were available, this relation was clearer for the EDC and BB and less clear for PL.

A second manner in which RFA might modulate the outputs of CFA is by altering the latency of the MEPs. As for peak intensity, we first examined the effect of the RFA stimulation on latency based on the averaged response obtained at each ISI. The modulation of the average peak latency (Δ*t*) was obtained by subtracting the latency of the aMEP_predicted_ peak from the latency of the aMEP_evoked_ peak for each ISI. If Δ*t* is negative, RFA conditioning decreased the peak latency, and, if Δ*t* is positive, RFA conditioning increased the MEP peak latency. As with ΔI, we found that the conditioning of RFA could have different effects on Δ*t* in different protocols and with different ISIs (see Fig. 1*C*). When looking at the Δ*t* values of all protocols (Fig. 8), it becomes clearer that RFA conditioning most often resulted in a decrease of Δ*t*, and this was more frequent when the C and T stimulations were delivered simultaneously or with a short ISI (0–5 ms). With longer ISIs (10 and 15 ms), there were more cases of RFA conditioning resulting in an increase of Δ*t*. In all cases, the effect of RFA conditioning on latency was small, being in the order of only a few milliseconds and <1 ms in 67% of cases. In contrast to ΔI, the pattern of modulation of MEP latency across ISIs was less clear and seemed to be different across muscles.

The repeated-measures ANOVA evaluating the relation between Δ*t* and ISI with the data combined from the three muscles for which sufficient MEPs were observed (EDC, PL, and BB) revealed a significant effect of ISI (*F* = 5.84, *P* = 0.002), and the within-subject contrast for the linear term was significant (F = 8.45, *P* = 0.006). Thus, RFA conditioning did have a tendency to shorten the latency of the MEP peaks when little time separated the C and the T stimulations and to increase latency when long ISIs separated the two stimuli. However, in contrast to ΔI, when looking at muscles individually, the effect of ISI on Δ*t* was only significant for EDC (*F* = 6.99, *P* = 0.001), and the within-subjects contrast for the linear term was significant for this muscle (*F* = 11.81, *P* = 0.003). Thus, only for EDC was there a significant linear relation between Δ*t* and ISIs.

A second analysis of peak latency based on individual trials was conducted. A population of predicted peak latencies was obtained from the sMEP_predicted_ trials and compared with the population of peak latencies obtained from the sMEP_evoked_ trials with a Wilcoxon rank sum analysis. Out of the 341 possible comparisons (see materials and methods), we found that C significantly affected the latency of the MEP in only 48 cases, suggesting that latency modulation by RFA conditioning was often weak. As for peak intensity, we separated the possible RFA effects on MEP latency across ISIs in four different categories. First, we found cases where RFA conditioning decreased the latency of the MEP of a muscle with at least one ISI but did not significantly increase the latency with any ISI (Group Earlier; *n* = 16 or 35.5%). Second, we found a few cases where RFA increased the latency of the MEP of a muscle with at least one ISI but did not significantly decrease the latency with any ISI (Group Later; *n* = 7 or 15.6%). Third, we found a few cases where RFA both decreased and increased the latency of the MEP of a muscle with different ISIs (Group Opposite; *n* = 3 or 6.7%). Finally, in the majority of cases, RFA conditioning did not significantly modulate the MEPs of a muscle with any of the ISIs (Not Sig; *n* = 19 or 42.2%).

**Fig. 9.**
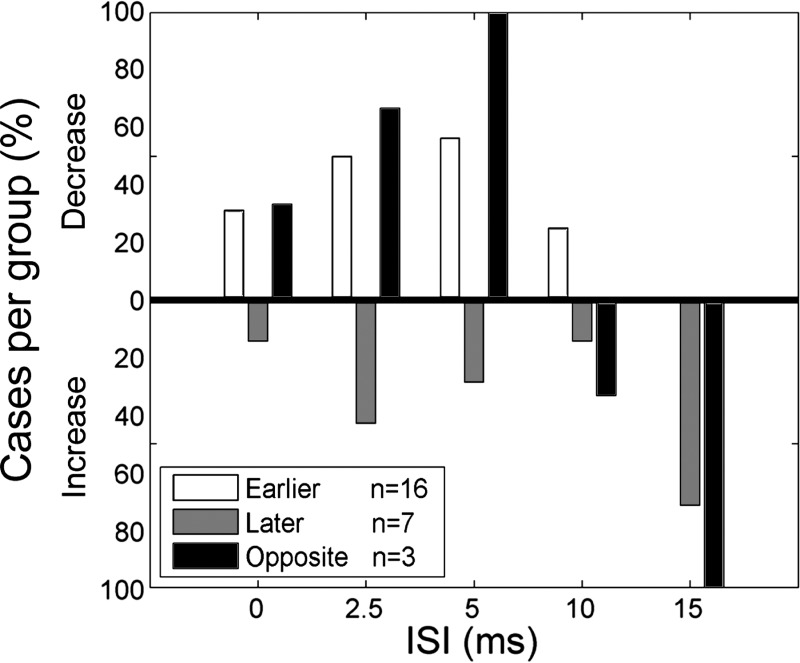
Categories of significant RFA conditioning effects on MEP peak latency. Results of the Wilcoxon rank sum analysis for peak latency. For a given protocol, we tested if the evoked MEP peak occurred significantly earlier or later than predicted MEP. As for peak intensity, the modulation of the RFA conditioning effects across ISIs was separated into 4 different groups [Group Earlier (*n* = 16), Group Later (*n* = 7), Group Opposite (*n* = 3), and Not Sig; *n* = 19]. Compared with the effects of RFA on peak intensity, there were more cases where RFA conditioning did not significantly modulate peak latency. The bar graph shows the proportion of significantly modulated cases for each group across the ISIs. For both Group Earlier and Group Opposite, the greatest proportion of RFA sites that significantly decreased the MEP peak latencies was with ISI5. For both Group Later and Group Opposite, the greatest proportion of RFA sites that increased the MEP peak latencies was with ISI15.

Figure 9 shows the distribution of the significant RFA effects across ISIs for each group. In Group Earlier, out of the 16 cases, eight had a significant decrease of MEP latency following RFA conditioning with ISI of 2.5 (50.0%) and nine with an ISI of 5 ms (56.3%). Significant decrease of latency was less common in this group when RFA and CFA were simultaneously stimulated (ISI0 = 31.3%) or following RFA conditioning with an ISI of 10 ms (25.0%). There was no case where RFA conditioning decreased latency with an ISI of 15 ms. In Group Later, increases of MEP peak latency were more common with ISI15 (71.4%) and ISI2.5 (42.9%) and less common with ISI5 (28.6%), ISI10 (14.3%), and ISI0 (14.3%). The three cases in Group Opposite followed a similar trend. RFA decreased MEP peak latency at shorter ISIs and increased it at longer ISIs. However, with the number of cases in each group being low, conclusions about the distribution for this analysis should be made cautiously.

### Relationship between MEP Peak Intensity and Peak Latency

As discussed above, we found a significant linear relationship between ΔI and ISI and between Δ*t* and ISI. Thus, the greatest increases of ΔI and the greatest decreases of Δ*t* were found with short ISIs. In contrast, with long ISIs, RFA showed the greatest inhibitory effects on MEP intensity and the greatest increases of Δ*t*. That led us to wonder if there was a relationship between the ΔI and Δ*t* or, in other words, if the two parameters changed simultaneously or independently. For all four muscles, there was a negative relationship between ΔI and Δ*t*, and ΔI was a significant predictor of Δ*t* (EDC, fixed effect estimate = −35.72, *P* < 0.001; PL, −21.87, *P* = 0.003; BB, −24.54, *P* = 0.001; TB, −180.13, *P* = 0.011). Thus, when RFA conditioning increased the MEP peak intensity, it tended to also decrease its latency. Oppositely, when RFA conditioning decreased the MEP peak intensity, it tended to increase its latency.

## DISCUSSION

In the present series of experiments, we studied the interactions between the two cortical forelimb motor representations in the rat. We use paired-pulse protocols where a C stimulus was applied in RFA, the proposed equivalent of a premotor area, simultaneously or before a T stimulus in CFA, the equivalent of M1. In some cases, RFA stimulation resulted in a substantial increase of the MEP, whereas in others, it completely abolished it. Different RFA sites had diverse effects on CFA outputs at different ISIs (facilitatory, inhibitory, or both). Stronger facilitatory effects were found when RFA and CFA were stimulated simultaneously, whereas stronger inhibitory effects were found when RFA stimulation preceded CFA stimulation by 10–15 ms. Whereas RFA conditioning had a much weaker effect on the latency than on intensity of the EMG responses, modulation of the two parameters was interrelated, with an inverse relationship between intensity and latency. Altogether, our data support that, as a premotor area in primates, RFA has powerful and complex interactions with CFA that can be used for the cortical control of arm and hand movements and to support motor recovery after lesions.

### Similarities between RFA and Premotor Areas of Primates

Several arguments have been proposed to support that RFA may be the equivalent of a premotor area. For example, whereas RFA is located in the medial agranular cortex, CFA is located in the lateral agranular cortex (AGl) (Wang and Kurata 1998). AGl has a broader layer V, with larger and denser cells, similar to M1 (Donoghue and Wise 1982). Compared with CFA, RFA has more connections with the insular cortex and sends more projections to the contralateral caudate putamen. RFA also receives more thalamic inputs from the ventromedial nucleus while thalamic projections to CFA mainly originate from the ventrolateral nucleus (Rouiller et al. 1993). Thus, the pattern of connections of RFA is more similar to that of premotor areas than M1 (Darian-Smith et al. 1990; Godschalk et al. 1984; Stepniewska et al. 1993).

Whereas both CFA and RFA have neurons with activity related to movement production (Hyland 1998), for similar behaviors in different contexts, the activity of RFA neurons is more dependent on the behavioral situation than the activity of CFA neurons (Saiki et al. 2014). Likewise in dorsal premotor cortex of monkeys, neural activity before movement is more complex than in M1 and appears to represent movements in an abstract manner, that is, more dependent on the task to be performed than on the arm that will do the movement (Cisek et al. 2003).

One way premotor areas can be involved in motor control is by modulating the motor outputs of M1 (Arai et al. 2012; Davare et al. 2008; Groppa et al. 2012). In monkeys, the interactions between ventral premotor cortex (PMv) and M1 have been most extensively studied with paired stimulation protocols (Cerri et al. 2003; Maier et al. 2013; Shimazu et al. 2004). These studies have shown that PMv has a powerful influence on M1 outputs to the arm muscles. In the present study, we showed that, similar to the effects of PMv on M1, RFA strongly modulates CFA outputs.

### Differences between RFA and Premotor Areas of Primates

There were also notable differences between the interactions of RFA with CFA we found in rats and the ones described between PMv and M1 in primates. One particular experiment looked at PMv and M1 interactions in ketamine-sedated monkeys (Cerri et al. 2003), a preparation similar to what we used in rats. PMv was found to have only facilitatory effects on M1 outputs, which was maximal when the stimulations were separated by 10–15 ms. In humans, PMv conditioning during precision grip follows a similar trend but with a maximal facilitation with ISIs of 6–8 ms (Davare et al. 2008). The wide range of modulatory effects of RFA on CFA we found, from potent facilitation to complete inhibition, appears more diverse than what was described for PMv in sedated (Cerri et al. 2003) and awake macaque monkeys during reaching and grasping movements (Prabhu et al. 2009). It is possible that the greater range of effects in rats was the result of the higher number of cortical sites tested compared with primate studies. Moreover, in the sedated preparation (Cerri et al. 2003), the effects of PMv were tested in only one intrinsic hand muscle. In rats, we studied the modulatory effect of RFA on CFA outputs to the forearm and arm muscles, thus making direct comparison of the results difficult. Nevertheless, it is tempting to suggest that RFA and PMv simply do not modulate outputs identically and that RFA neurons may have a wider range of influence than PMv neurons have on M1 outputs. If the motor cortex evolved to differentiate several subdivisions in primates, perhaps RFA in the rat does not play a role that is equal to any given premotor area, but may rather combine several premotor functions.

The linear relationship between ΔI and ISI in rats, with the greatest facilitatory effect with ISI of 0 ms, is different from the relationship found for PMv in monkeys and humans and does raise the possibility that some of the interactions occur at different sites in the two species. The maximal facilitatory effects found with simultaneous stimulation of RFA and CFA are unlikely to be simply explained by current spread between the two electrodes. We verified current spread for each pair of electrodes using the relationship *I* = *kr*^2^ (Stoney et al. 1968), where *I* is current (in mA), *r* is the radius of current spread (in mm), and *k* is a constant (1,292 mA/mm^2^). In all cases, the estimated radius of current from the C and T electrodes did not overlap. The closest pair of C and T electrodes had a 0.45-mm buffer between the radii of estimated current spread, and, on average, pairs of electrodes had 1.7 mm between the estimated radii. Moreover, the relationship between the MEP peak intensity with ISI of 0 ms and the distance separating the C and T electrodes was not significant (Pearson's *r* = −0.09; *P* = 0.43). We conclude that the facilitatory effects with ISI0 were the result of interactions of the two populations of stimulated neurons.

One possibility is that facilitatory interactions between RFA and CFA in rats mainly take place at a subcortical level, for example, in the brain stem, spinal cord, or both. Projections to the spinal cord and brain stem from RFA and CFA are comparable (Rouiller et al. 1993) and could favor subcortical convergence. In monkeys, the maximal modulatory effects of PMv being at longer ISIs rather suggest that the interactions occur serially, in M1 (Cerri et al. 2003). Corticospinal projections from PMv are much sparser than from M1 and mainly reach upper cervical levels (Borra et al. 2010; He et al. 1993). The indirect access to lower spinal cord levels may favor the serial influence of PMv through M1. Further supporting this hypothesis, inactivation of M1 reduces the capacity to evoke hand movements from PMv (Schmidlin et al. 2008).

In the context of movement production, the predominant inhibitory effects of RFA with longer ISIs suggest that activation of RFA neurons earlier during preparatory stages mainly decreases the excitatory state of neurons within the motor pathway from CFA to forearm muscles. Perhaps this inhibition would preshape a specific “muscle field” that can generate the intended movement. The predominant facilitatory effects of RFA with shorter ISIs supports that, at the stage of sending the outputs from CFA, RFA neurons would then reinforce outputs from CFA neurons within the pathway to the selected muscles. However, it is worth nothing that cortical interactions can be affected by sedation or the task being carried out (Cerri et al. 2003; Davare et al. 2008; Prabhu et al. 2009). Therefore, a more complete understanding of the effects RFA on CFA outputs will warrant additional studies in awake-behaving rats.

### The Effect of Test Stimulations in CFA on MEPs

In rats, spinal recordings of MEPs evoked from epidural cortical stimulations show multiple I waves at different latencies (Ryder et al. 1991; Shiau et al. 1992). The peak latency of the first wave carried through the corticospinal pathway occurs ∼13 ms after stimulation and the peak of the second wave at approximately two times the latency (Shiau et al. 1992).

In our study, we found that forearm muscle MEP peak latencies were ∼16–17 ms following single ICMS pulses in CFA, and there was little evidence of later peaks (see Fig. 3). These values are slightly longer, but comparable, to the ones reported in another study where latencies were calculated from forearm muscle MEPs following single pulse ICMS (onset latencies of 9.73 ± 1.82 ms and a rise time to peak of 3.43 ± 1.75 ms) (Liang et al. 1993). Nevertheless, latencies we obtained and the ones reported by Liang and collaborators (1993) are both shorter than the ones expected from the second corticospinal wave (Shiau et al. 1992) and suggest that forearm muscle MEPs resulting from single ICMS pulses in CFA result from the first wave.

With the use of intracortical stimulations, it has been reported that intensities above 0.6 mA can induce faster responses, as short as 3.6 ms, presumably because of the spread of the current to subcortical structures (Liang et al. 1993). Using relatively low current intensities in the present study (<300 mA), we did not find MEP peak values lower than 13 ms. Additionally, the estimation of current spread resulting from a maximal T stimulation of 300 μA is <0.5 mm, too short to expect activation of extrapyramidal descending pathways. Thus, the MEPs evoked by the T stimulations most likely result from the stimulation of cortical neurons in CFA.

### Similar Simultaneous Effects on Recorded Muscles of the Arm

Stimulus-triggered averaging of EMG using single pulses in RFA and CFA in rats has shown that neurons in both areas can simultaneously cofacilitate several proximal and distal muscles (Liang et al. 1993). In our study, we only recorded two distal and two proximal muscles. Stimulation of CFA (T stimulation only) simultaneously evoked EMG activity in all four muscles for 50% of cortical sites and cofacilitated three of the four muscles (all but TB) for the remaining 50%. With paired-pulse stimulations, we found that RFA conditioning can affect the entire muscle field of a CFA cortical site but can also affect only some muscles within the field. We found comparable incidence of cases where RFA affected one, two, three, or all four muscles within the field of CFA neurons. Hence, it seems that RFA neurons can have both interactions with a specific muscle within the field of a CFA site or have a generalized effect on its entire field.

### Effect of RFA Conditioning on the Latency of CFA Outputs

When looking at the average MEPs for the different protocols (Fig. 8), RFA conditioning tended to decrease the latency with short ISIs and increase it with long ISIs. However, modulation of latency was typically small (<1 ms) and only significant in 14% of cases. Whereas the effect of RFA conditioning on latency could be better quantified using surface recording of spinal volleys or intracellular recording of motoneurons (Maier et al. 2013; Shimazu et al. 2004), we did find cases where RFA conditioning changed latency by several milliseconds. It is tempting to conclude that the weak effect we found was not because of methodological limitations but instead that most RFA neurons only mildly affected CFA output latency. Thus, while most RFA neurons strongly affected CFA output intensity, they had only mild effects on latency. Nonetheless, ΔI and Δ*t* were interrelated such that when an RFA site increased a CFA site output's intensity, it tended to shorten its latency as well. In contrast, when an RFA site decreased the intensity of a CFA output, it increased its latency. These results are consistent with the notion that increased synaptic excitation drives the neurons closer to threshold, thereby simultaneously decreasing latency.

### General Conclusions

In spite of some unresolved issues regarding phylogenetic considerations, our data show that, like PMv in primates, RFA can shape cortical outputs in diverse and dramatic ways to evoke activity in forearm muscles and play a key role for the cortical control of movement. Lesions of the motor cortex in rats induce severe and lasting deficits and reveal the crucial contribution of cortical motor areas in the control of skilled hand and arm movements (Barth et al. 1990; Gharbawie et al. 2005). Similar to a premotor area, RFA is particularly well positioned to participate in motor recovery following cortical injury.

## GRANTS

This work was supported by Natural Sciences and Engineering Research Council of Canada (RGPIN/402663-2011) and Heart and Stroke Foundation Canadian Partnership for Stroke Recovery Grants to N. Dancause. N. Dancause is supported by a Canadian Institutes of Health Research New Investigator Salary Award.

## DISCLOSURES

No conflicts of interest, financial or otherwise, are declared by the authors.

## AUTHOR CONTRIBUTIONS

Author contributions: J.E.D., B.T., and S.Q. performed experiments; J.E.D. and S.Q. analyzed data; J.E.D. and N.D. prepared figures; J.E.D. and N.D. drafted manuscript; J.E.D., B.T., S.Q., and N.D. approved final version of manuscript; S.Q. and N.D. interpreted results of experiments; N.D. conception and design of research; N.D. edited and revised manuscript.
